# Effects of Cell-Attachment and Extracellular Matrix on Bone Formation *In Vivo* in Collagen-Hydroxyapatite Scaffolds

**DOI:** 10.1371/journal.pone.0109568

**Published:** 2014-10-16

**Authors:** Max M. Villa, Liping Wang, David W. Rowe, Mei Wei

**Affiliations:** 1 Department of Materials Science & Engineering, University of Connecticut, Storrs, Connecticut, United States of America; 2 Center for Regenerative Medicine and Skeletal Development, Department of Reconstructive Sciences, School of Dental Medicine, University of Connecticut Health Center, Farmington, Connecticut, United States of America; Texas A&M University Baylor College of Dentistry, United States of America

## Abstract

Cell-based tissue engineering can be used to replace missing or damaged bone, but the optimal methods for delivering therapeutic cells to a bony defect have not yet been established. Using transgenic reporter cells as a donor source, two different collagen-hydroxyapatite (HA) scaffolds, and a critical-size calvarial defect model, we investigated the effect of a cell-attachment period prior to implantation, with or without an extracellular matrix-based seeding suspension, on cell engraftment and osteogenesis. When quantitatively compared, the in-house scaffold implanted immediately had a higher mean radiopacity than in-house scaffolds incubated overnight. Both scaffold types implanted immediately had significantly higher area fractions of donor cells, while the in-house collagen-HA scaffolds implanted immediately had higher area fractions of the mineralization label compared with groups incubated overnight. When the cell loading was compared *in vitro* for each delivery method using the in-house scaffold, immediate loading led to higher numbers of delivered cells. Immediate loading may be preferable in order to ensure robust bone formation *in vivo*. The use of a secondary ECM carrier improved the distribution of donor cells only when a pre-attachment period was applied. These results have improved our understanding of cell delivery to bony defects in the context of *in vivo* outcomes.

## Introduction

Cell-based bone tissue engineering holds promise to supplement or replace the limited supply of autologous bone for bone grafting procedures. Osteoprogenitors can be sourced from the bone marrow, expanded *in vitro*, and seeded to a scaffold to form a bone graft. Several animal studies using a scaffold combined with bone marrow stromal cells (BMSCs) have shown encouraging results healing bone defects. [1]–[3] This approach was also tested in a few human patients, and appeared to form new bone when viewed radiographically. [4], [5] However, there has been no large-scale clinical trial of cell-based bone tissue engineering as of yet, and this approach has lagged behind growth factor based-approaches. [6] Negative side effects have been observed in connection with the delivery of a single morphogenetic factor, which requires very high doses to be effective. [7] By contrast, cells produce hundreds of factors during healing, and a cell-based approach could sidestep limitations regarding side effects associated with supraphysiological doses of growth factors. However, several issues remain before effective bone formation using culture-expanded osteoprogenitors becomes a clinical reality. These include optimizing cell delivery to a site of bone injury.

A number of methodologies have been proposed to optimize cell seeding efficiency [8]–[11] and *in vitro* culture conditions. [11]–[13] However there is limited *in vivo* evidence [14] describing which approaches are most effective at healing a bony defect. Presently in the clinic, whole bone marrow aspirate can be added to a scaffold material at the time of implantation. While this approach avoids any *in vitro* manipulation, and the associated time and cost, the progenitor number is very low in the bone marrow [15]. Previously, progenitor number has been correlated to the volume of mineralized callus formed after implantation to a fracture nonunion, and in some cases low progenitor number led to suboptimal healing. [16] By contrast, *in vitro* expansion can provide large numbers of osteoprogenitors and therefore increase the therapeutic power of a cell-based approach. Culture expansion is used here to ensure that large numbers of osteoprogenitors are delivered to the bony injury. Nonetheless, the degree of cell attachment, distribution, and phenotype in a cell-seeded scaffold is largely unknown. Therefore the construct initial conditions (cell attachment, number, viability, phenotype) require continued examination in terms of *in vivo* outcomes. Examination of a tissue-engineered construct prior to implantation can be a useful quality control checkpoint when used in the clinic. [17] Previous work in the mouse has shown an upper limit on the length of time osteoprogenitors can be cultured *in vitro* and still produce bone *in vivo*. [14] In this case, a length of about six to eight days corresponded to maximal bone formation, due to increasing osteoblastic differentiation beyond this period. Similarly, it has also been suggested that when human cells are used for bone tissue engineering, an osteoprogenitor phenotype, rather than differentiated osteoblasts, may lead to better *in vivo* osteogenesis [18].

**Figure 1:**
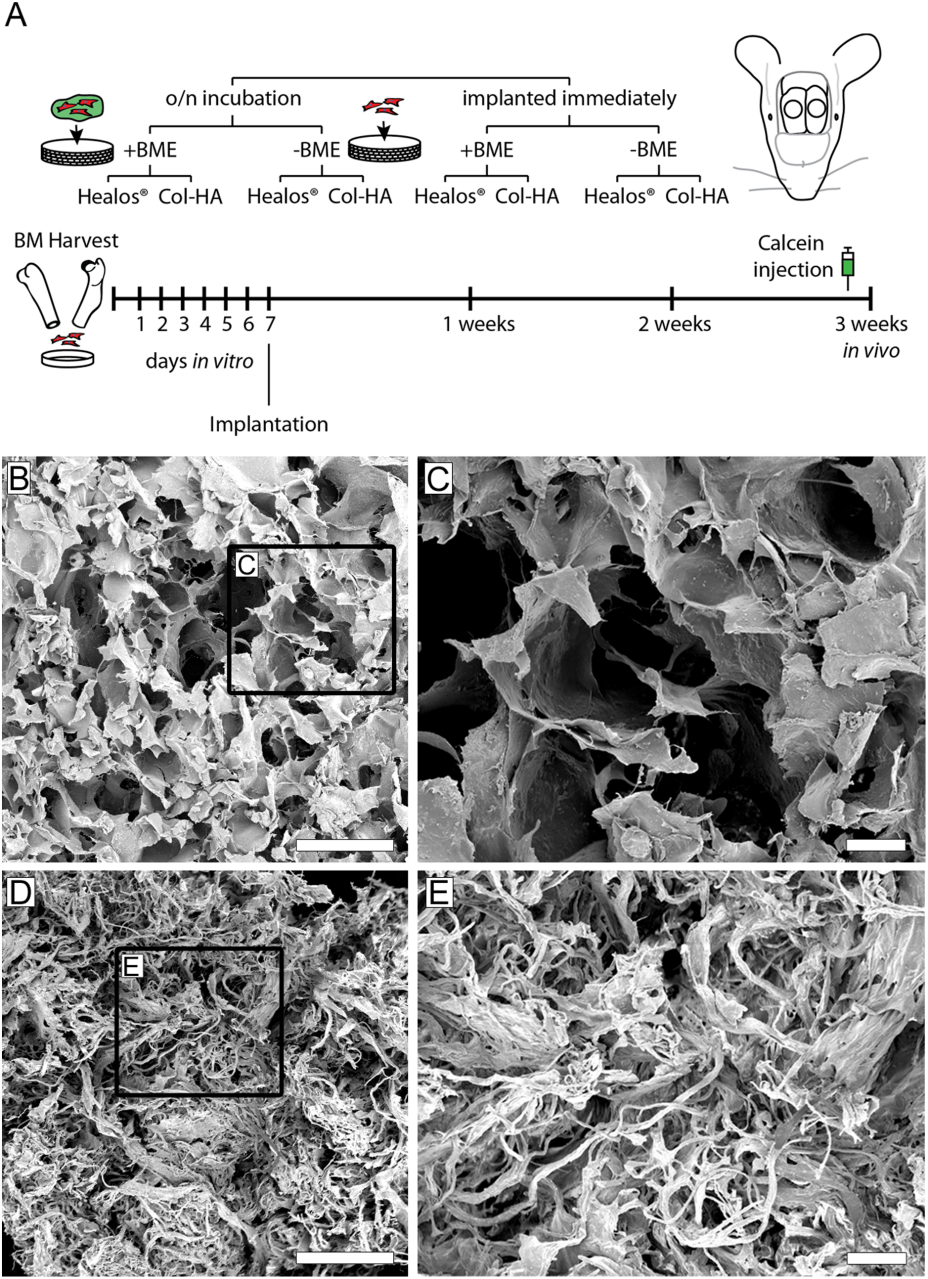
Experimental design and scaffold morphology. (A) Schematic of Experimental Design. Osteoprogenitors from the bone marrow were expanded *in vitro* before seeding to collagen-hydroxyapatite scaffolds and implanted in critical size calvarial defects. To label areas of active mineralization, calcein was injected intraperitoneally one-day prior to euthanization at three weeks post-implantation. (B) Electron micrograph of the Col-HA scaffold showing cellular morphology. Scale bar is 500 µm. (C) Enlarged inset from (A). Scale bar is 100 µm. (D) Electron micrograph of Healos scaffold. Scale bar is 500 µm. (E) Enlarged inset from (D). Scale bar is 100 µm.

Cell attachment is linked to cell viability through integrin binding [19] and may enhance the survival of implanted cells. [20] We sought to evaluate the effect of a cell-attachment period prior to implantation on bone formation. To examine this question, bone formation within scaffolds seeded at the time of implantation was compared to scaffolds seeded and incubated overnight to allow complete cell attachment prior to implantation (Fig. 1). Implanted constructs were examined with radiography and histology after three weeks *in vivo.* If cell attachment prior to implantation improves *in vivo* outcomes, this would have implications for the clinical delivery of cells, which are currently seeded to a scaffold at the time of implantation. The vast majority of cells are not attached to the scaffold within minutes of seeding. [21] More critically, it is unclear if the *in vivo* wound microenvironment promotes or hinders cell attachment.

We also sought to evaluate the effect of an extracellular matrix (ECM) carrier material on bone formation, possibly helping to hold cells in position after seeding, and/or conferring benefits such as synergistic signaling by the ECM. [22] The ECM performs several critical roles in signal presentation and transduction. [22]–[26] Recent reports have indicated that the ECM promotes cell retention, survival, and differentiation. [20], [27], [28] Here we evaluated the use of a reduced growth factor formulation of basement membrane extract (BME) gel (Cultrex, Trevigen Inc., also sold as Matrigel) as a cell suspension to seed osteoprogenitor cells to a scaffold. BME gel consists mainly of laminin, entactin, and collagen IV. Four hundred and eighty unique proteins were identified within growth factor-reduced BME, representing a complex matrix of extracellular, binding, catalytic, and regulatory proteins [29].

The goals of this work were to (i) examine the effect of a cell attachment period prior to implantation on bone formation, and (ii) to assess the effect of a complex ECM as a secondary delivery carrier on bone formation. We evaluated these conditions in two different collagen-hydroxyapatite scaffolds, Healos (DePuy) and an in-house scaffold, here denoted Col-HA. Identifying the optimal conditions for cell-delivery should improve the efficacy and repeatability of cell-based bone tissue engineering.

## Materials and Methods

### Scaffold fabrication and sterilization

To fabricate the in-house scaffold, type-I collagen was first derived from rat tail tendons following Rajan *et al*. [30] A collagen-hydroxyapatite composite was then formed by self-assembly of collagen fibers in the presence of precipitating hydroxyapatite from a modified simulated body fluid (m-SBF) [31]. Briefly, the collagen solution was adjusted to 2.5 mg/mL by a two-fold dilution in sterile ultrapure water at 4°C. To make a 200 mL solution of collagen-containing m-SBF, the following salts were added in the order they appear to the 2.5 mg/mL collagen solution: 1.080 g NaCl, 0.142 g K_2_PO_4,_ 0.062 g MgCl_2_, 2.400 g (4-(2-hydroxyethyl)-1-piperazineethanesulfonic acid) HEPES, 0.175 g CaCl_2_, and 0.294 g NaHCO_3_. While kept cold to prevent collagen fibrillogenesis, the pH of the solution was adjusted to 7.0 with sodium hydroxide solution and then transferred to a water bath at 40°C for 24 hours to allow simultaneous precipitation of hydroxyapatite and collagen fibrillogenesis. The gel-precipitate was centrifuged at 11,000 g and 4°C for 12 min. The supernatant was discarded and the pellet was lyophilized (FreeZone 12L, Labconco). The collagen-HA precipitate was reconstituted with water at a concentration of 100 mg/mL, briefly homogenized to obtain a uniform slurry, and frozen in a polystyrene culture dish from room temperature to −40°C at a cooling rate of −0.37°C/min. Following drying, the scaffold was immersed in a solution of 20 mg/mL EDC [1-ethyl-3-(3-dimethylaminopropyl) carbodiimide hydrochloride] for 24 hours at 4°C to covalently crosslink the collagen fibers. The scaffold was then rinsed in a solution of 5% (w/w) glycine in sterile water for an overnight period in order to block unreacted EDC. Crosslinking was followed by three sequential rinses in sterile water for 15 minutes each at 4°C. Finally, rinsed scaffolds were freeze-dried, cut to a thickness of ∼500 µm with a milling machine and punched to a diameter of 3.5 mm. Scaffolds were terminally sterilized with a 24-hour cycle of ethylene oxide gas (Anprolene AN74i, Anderson Products). The Healos material was received sterile. Healos is a lyophilized bovine type-I-collagen sponge with high porosity and a pore size ranging from 4–200 µm, coated with a thin layer of calcium phosphate.

### In vitro culture of bone marrow osteoprogenitors

Mouse BMSCs were isolated from the femur and tibia of OsterixCRE/Ai9 B6 animals. Osterix is a critical regulator of osteoblast differentiation. [32] The combination of the OsterixCRE and Ai9 [33] transgenes activate the expression of the TdTomato (red) fluorescent protein following Osterix expression. This reporter construct activates the production of TdTomato when a cell expresses Osterix, and continues expression of TdTomato in subsequent daughter cells. Thus, following three weeks of *in vivo* implantation, the TdTomato signal will label osteoprogenitors, committed osteoblasts, and osteocytes. Bone marrow cells from femur and tibia were collected by a centrifugation method. Briefly, interlocking filtration column and collection tubes were modified by removing the filter and autoclaved. Femur and tibia were cut in half and placed cut end down in the top tube. The bones were spun at 3100 g for 2 minutes, ejecting the bone marrow cells into the lower collection tube containing 100 µL of PFE (98% PBS, 2% FBS, 2 mM EDTA) to prevent clotting. Bone marrow cells were flushed through a 70 µm cell strainer (BD Biosciences) and re-suspended in α-MEM. Cells were then added to 100 mm culture dishes in warm α-MEM (Gibco) containing 10% fetal bovine serum and 1% penicillin-streptomycin. To allow hematopoietic cells to contribute to the expansion of osteoprogenitor cells, the culture medium was changed four days after seeding the bone marrow pellet. Two different types of scaffolds (Healos and Col-HA) were seeded the day before implantation or incubated overnight, with or without BME gel as a seeding suspension, resulting in eight groups (n = 3). The sample hierarchy is shown schematically in Fig. 1A. Cells were either seeded in a suspension of growth factor reduced Basement Membrane Extract (BME, 15 mg/mL in PBS, Trevigen) or in a suspension of α-MEM. The cell suspension was made by adding 15 µL of culture medium, or cold BME, to a cell pellet containing 1.0×10^6^ cells, resulting in a concentration of 6.66×10^7^ cells/mL. Prior to seeding, adherent BMSCs were trypsinized and seeded dropwise on top of either the dry Col-HA scaffold or Healos. After seeding, cells were allowed to settle for 30 minutes before an additional one mL of warm culture medium was added to the culture well. On the day of implantation, samples denoted ‘implanted immediately’ were seeded with 1.0×10^6^ cells in 15 µL of culture medium, or BME, and implanted in less than one minute after seeding.

### In vivo mouse model of bone repair

On the day of implantation, nod *scid* gamma immunodeficient mice were anesthetized with a ketamine (135 mg/kg) – xylazine (15 mg/kg) blend and two 3.5 mm diameter critical-size defects were made in the right and left parietal lobe using a bur trephine (RAL #229-030, Benco Dental). Extreme care was taken to prevent damage to the dura mater beneath the calvarium. Seeded constructs were placed in the defects, alternating the order of scaffold type in the left and right hole, for each of the 12 animals. Following closure of the scalp with resorbable sutures, animals were given postoperative analgesic (bupronephrine, 0.08 mg/kg). One day prior to euthanization at three weeks post-implantation, host mice were injected intraperitoneally with calcein to mark surfaces of active mineralization. All procedures used in this study were approved by the UConn Health Center Institutional Animal Care and Use Committee (IACUC).

### Image acquisition and analysis

After three weeks of implantation, animals were euthanized, and the extracted calvarium were fixed in 10% formalin overnight. The following day, samples were placed in a 30% sucrose solution, while kept cold and in the dark for another overnight period. Radiographs of calvarium were acquired with a digital X-ray system (MX20, Faxitron). The samples were then prepared for histology by trimming the calvaria to the defect regions, and embedding each sample in Cryomatrix (Thermo). Three sections were cut from each calvarium using a cryostat (Leica) and tape to transfer sections to a glass slide (Cryofilm Type2C, Section-Lab). All sections were imaged with a 10× objective on a fluorescent microscope (Axio Scan.Z1, Zeiss) equipped with stage automation and an LED light source (Colibri.2, Zeiss).

Bone formation was quantified from radiographic images by selecting a region of interest (ROI) surrounding only the defect area and calculating the mean pixel intensity using FIJI. [34] To quantify the area fraction of donor cells, calcein label, and darkfield signal from histological sections for each group, an ROI outlining the defect area was manually drawn for each image and saved using FIJI. A threshold was then applied to each channel, and the area fraction was calculated from the total defect area. To process the images as a batch and ensure the same threshold was consistently applied, a macro was written for FIJI.

To determine the position of each cell pixel relative to the outside edge of the implant, a distance mapping technique [35] was performed. Briefly, a distance transformation was applied to the defect region, coding pixel intensity according to pixel distance from the outer perimeter of the defect (i.e. intensity of 20 corresponds to 20 pixels from the perimeter). Then a threshold was applied to the cell image and converted to binary (0 for background, 255 for cells) to be used as a mask when applied to the distance map. The image calculator tool was used to find the minimum value between the distance map and the cell mask. Since the cell pixels are always higher than the map pixels in this case, the resultant image contains distance-coded information at each cell pixel. Noting the spatial conversion from pixels to microns, a histogram of the resultant image yields the number of cell pixels as a function of distance from the outside edge for each section.

### Statistical analysis

Quantitative data is presented as the mean ± standard deviation (error bars and blue bars). In some cases 95% confidence intervals (light red bars) are also included. A two-sample independent t-test was used to determine significance between groups. P-values less than 0.05 (both tails) were considered statistically significant and are indicated with an asterisk. P values less than 0.01, 0.001, and 0.0001 were indicated with two, three, and four asterisks, respectively.

### Permeability Measurement

The permeability, k (m^4^/Ns), of Healos and Col-HA scaffolds was measured with water and a custom flow cell using the following equation found in reference [36]:
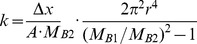
where, Δ*x* = scaffold length (m), A = scaffold cross-sectional area (m^2^), M_B1_ = mass flow rate without scaffold (g/s), M_B2_ = mass flow rate with scaffold (g/s), and r = radius of the outlet (m).

## Results

### In vitro examination of expanded cells

**Figure 2:**
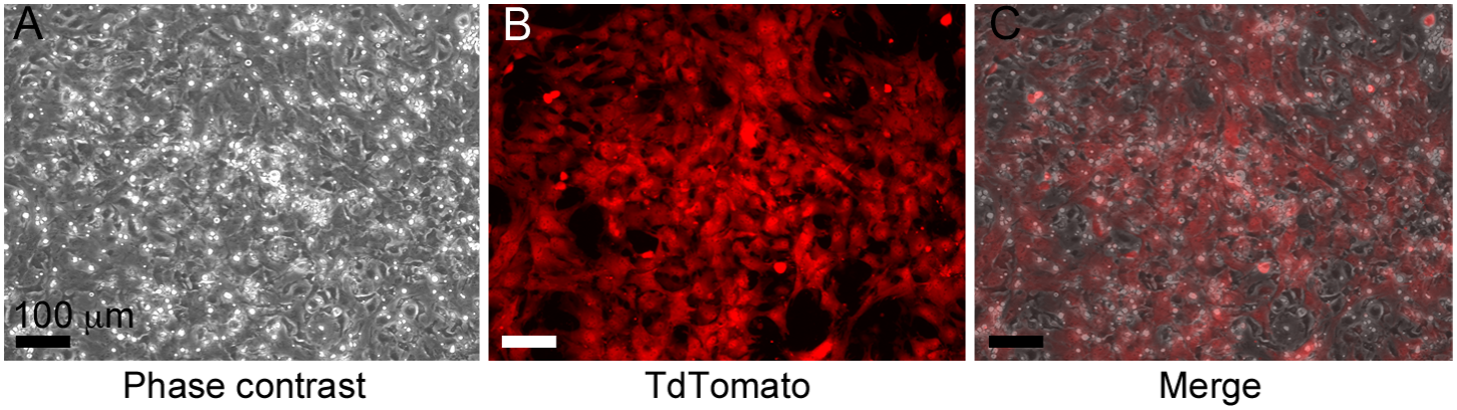
Osteoprogenitor cells following six days of *in vitro* expansion. (A) Phase contrast showing near confluent cell culture, (B) TdTomato reporter driven by OsterixCRE and (C) Merged image of (A) and (B) indicating a large fraction of cultured cells are osteoprogenitors.

Four days after plating whole bone marrow, colonies of fibroblastic cells had begun to appear in the culture dish. Following six days *in vitro*, colonies had proliferated and become confluent. When examined using a fluorescent microscope, fibroblastic cells were positive for the TdTomato osteoprogenitor marker (Fig. 2), indicating that large numbers of osteoprogenitor cells were present in the culture dishes prior to scaffold seeding.

### Radiographic evaluation of calvarial defects following three weeks in vivo

**Figure 3:**
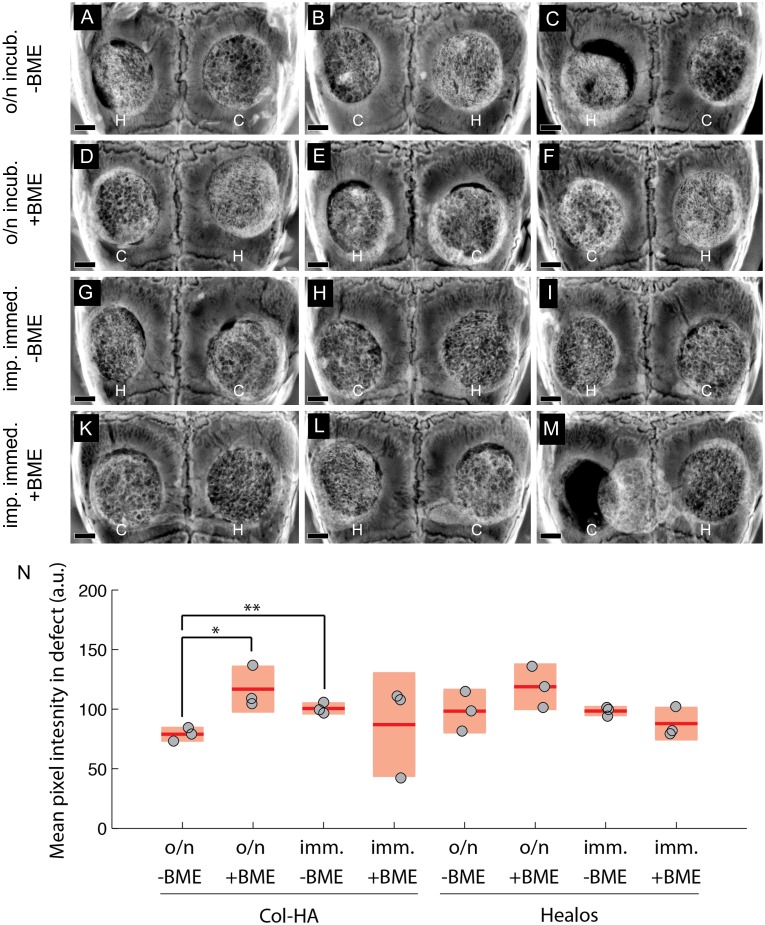
Radiographs of cell-scaffold constructs after 3 weeks *in vivo*. (A)–(M) H and C denote Healos and Col-HA scaffolds, respectively. Scale bars are 1 mm. (N) Quantitation of radiographs. Light red bars indicate 95% confidence intervals and blue bars indicate one standard deviation.

Three weeks after implantation, calvaria were harvested to examine the implants. By radiographic discrimination (Fig. 3), it appeared that all samples contained areas of radiopacity indicative of mineralized tissue. Both scaffolds contain hydroxyapatite (∼30% by weight), however if collagen-HA scaffolds seeded with cells undergo mineralization *in vivo*, a clear increase in radiopacity is observed (an example radiographic progression is shown in Fig. S1). Two of the 24 scaffolds had moved from the defect region (Fig. 3C and 3M), however the remaining implants appeared in good contact with the surrounding host bone. Quantification of the radiographic images showed significantly greater radiopacity in the Col-HA samples seeded immediately compared with the Col-HA samples provided an overnight incubation (Fig. 3N). When BME gel was used as the seeding medium during overnight incubation of Col-HA samples, the mean radiopacity was greater than Col-HA samples incubated overnight without BME gel (Fig. 3N). Histological examination was performed to further examine implant osteogenesis.

### Histological evaluation of calvarial defects following three weeks in vivo

**Figure 4:**
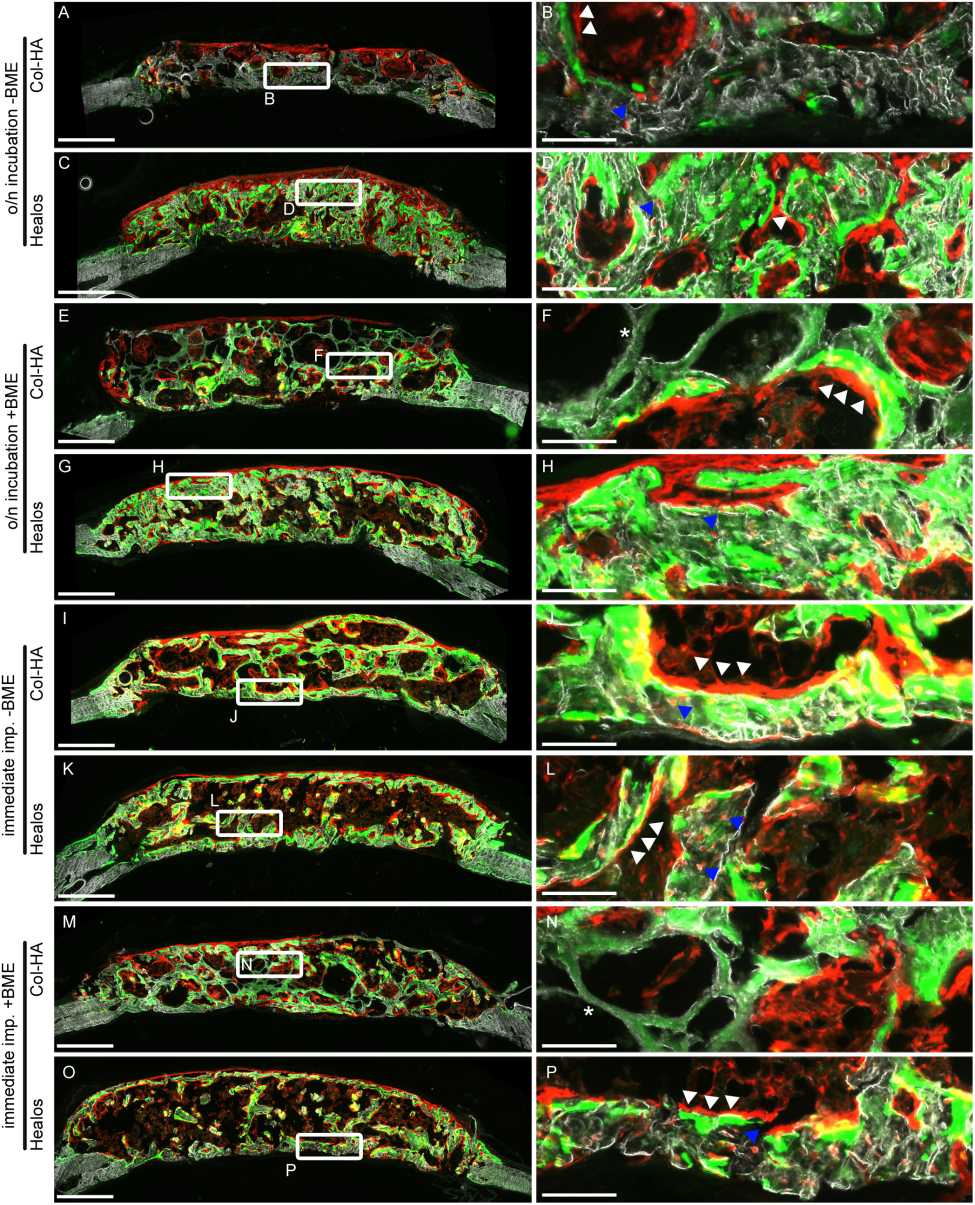
Histological sections of defects following 3 weeks of *in vivo* implantation. (Left column) Defect wide view of scaffolds and mineralized tissue (darkfield channel), +TdTomato donor cells (red), and the mineralization label (green, calcein). Scale bars are 500 µm. (Right column) Magnifications of left column. Blue arrows indicate +TdTomato donor cells embedded in bone. White arrows indicate +TdTomato donor cells overlying mineral label. Scale bars are 100 µm.

To verify bone formation and determine the distribution of donor cells in the implants (marked by a TdTomato fluorescent reporter, Fig. 2), histological sections of the calvaria were generated (Fig. 4). Histological examination of the implants showed bone formation in several samples, indicated by donor cells embedded in a mineral phase (Fig. 4, right column, blue arrows). Modest bone formation was found in the Col-HA groups incubated overnight (Fig. 4A and 4E). The Healos samples appeared to contain more bone and less scaffold than the Col-HA samples, which contained areas of scaffold still intact (Fig. 4F and 4N, white astericks). Several pores in the Col-HA scaffolds did not contain donor cells (Fig. 4A, 4F, and 4N), suggesting limited cellular invasion.

The Healos samples without incubation (Fig. 4K and 4O) had larger marrow spaces than the Healos samples with an overnight incubation prior to implantation (Fig. 4C and 4G). Marrow spaces also contained cells of donor origin (Fig. 4P and 4L). Fewer marrow niches appeared to have emerged in the Col-HA samples (Fig. 4A, 4E, and 4M).

Donor cells were found on the surface of the mineral label, pointing to their contribution to bone formation in the defects (Fig. 4, white arrows). Mineral label was present in all samples, indicating the label was able to diffuse into the tissue by systemic delivery. Some regions of the Col-HA scaffold appeared to take up the mineral label as a weak nonspecific stain (Fig. 4F and 4N, white astericks) rather than a distinct line characteristic of an active mineralizing surface.

### Quantitative histomorphometry following three weeks in vivo

**Figure 5:**
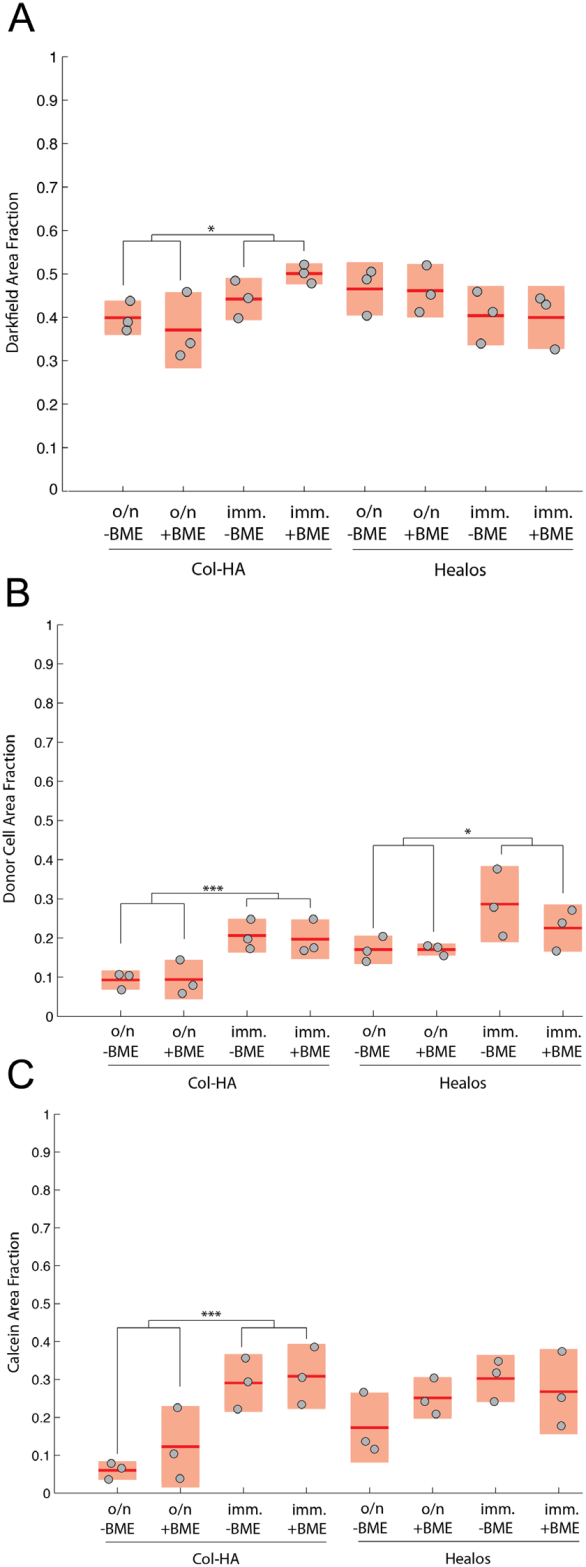
Quantitative histomorphometry of darkfield, donor cell, and calcein channels. (A) Quantification of the darkfield area fraction in the defect area. (B) Quantification of donor cell area fraction using TdTomato signal. (C) Quantification of mineralizing surface using the calcein mineralization label. Light red bars indicate 95% confidence intervals and blue bars indicate one standard deviation.

To quantitatively compare the experimental groups, a histomorphometric analysis was performed. Analysis of the darkfield channel (shown as the grayscale channel in Fig. 4) indicated that the Col-HA groups implanted immediately had a higher mean darkfield area fraction than the Col-HA groups incubated overnight (Fig. 5A). It should be noted that the darkfield signal contains areas of bone and scaffold, as shown in Fig. 4E. When the donor cell area fractions were examined, both Healos and Col-HA samples wherein cells were implanted immediately had significantly higher area fractions of donor cells than the same scaffold type given an overnight attachment period (Fig. 5B). The Col-HA samples implanted immediately had a higher mean calcein area fraction compared with the Col-HA samples incubated overnight (Fig. 5C). By contrast, the mean donor cell and calcein area fractions were very similar whether or not BME gel was applied. As a whole, histomorphometric analysis of the darkfield, donor cell, and calcein area fractions supported the notion that immediate implantation led to an defect with more donor cells and mineralizing surfaces compared with samples incubated overnight.

**Figure 6:**
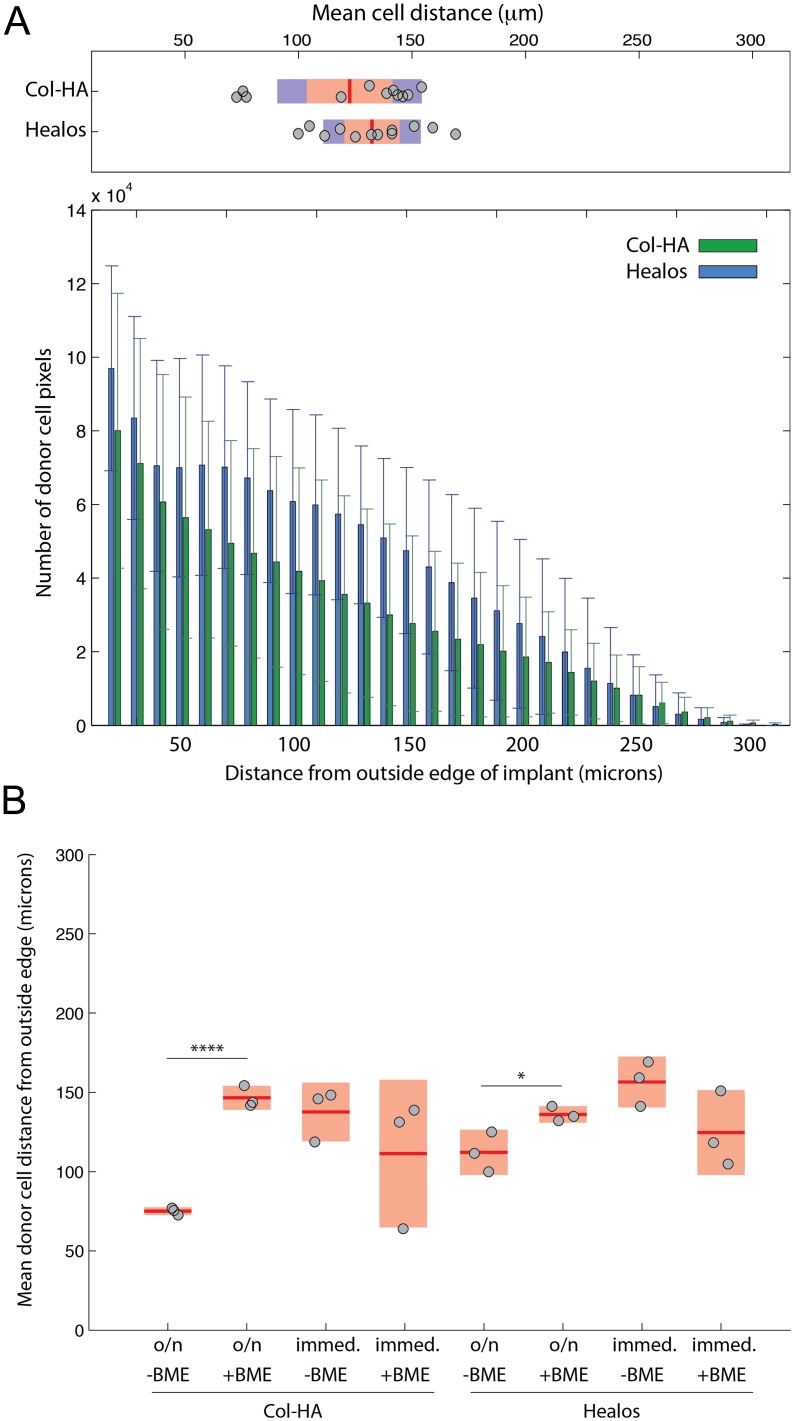
Donor cell distribution in the scaffolds. (A) Comparison of donor cell distribution from the outside edge of the implant for Healos and Col-HA. Error bars indicate one standard deviation. Plot above shows a comparison of the means for each sample according to scaffold type. (B) Comparison of the mean donor cell distance from the outside edge of the implant. Light red bars indicate 95% confidence intervals and blue bars indicate one standard deviation.

To examine the effect of scaffold type and delivery method on the distribution of donor cells in the implant, a distance analysis was performed. For each defect, a distribution of donor cells as a function of distance from the outside edge of the implant was generated. When viewed by scaffold type, the donor cell distribution in the Healos samples was not significantly deeper when compared to the Col-HA samples (Fig. 6A). Interestingly, the groups incubated overnight had significantly deeper mean cell penetration when BME gel was included, regardless of scaffold type (Fig. 6B). The inclusion of BME gel may increase cell penetration only if cells are incubated in the scaffold overnight.

### Permeability measurement of scaffolds used in vivo

When the permeability of the two scaffolds was compared, the Healos sample was more permeable than the Col-HA sample by three orders of magnitude, 4.2±3.4×10^−9^ and 2.9±3.0×10^−12^ m^4^/N-s, for Healos and Col-HA, respectively.

## Discussion

We examined if an *in vitro* cell attachment period before implantation, and/or an ECM-based delivery suspension, would improve donor cell survival and bone formation *in vivo*. The outcome of the experiment presented here suggests that immediate implantation improves donor cell delivery and scaffold mineralization; likely due to a higher number of cells implanted in this case (Fig. S3). Immediate loading corresponded to a higher mean radiopacity in the Col-HA scaffolds compared with Col-HA scaffolds provided with an overnight incubation (Fig. 3N). Similarly, the donor cell area fraction was significantly higher for both types of scaffolds implanted immediately compared with the same scaffold type incubated overnight. Finally, the mean area fraction of the mineralization label was significantly higher for the Col-HA scaffolds implanted immediately compared with Col-HA scaffolds given an overnight incubation (Fig. 5C). This suggests that cell attachment and survival may not be hampered by the *in vivo* microenvironment of a fresh bone injury. Furthermore, immediate seeding and implantation is faster, requires less cell manipulation, and in this study, enabled the delivery of significantly higher numbers of donor cells (Fig. S3). The efficient use of a precious cell source would also be advantageous. An increase in radiopacity was not found in the Healos groups seeded immediately, possibly because of bone remodeling characteristic of a more advanced healing stage [37]. This was evidenced by the large marrow spaces found in Fig. 4K and 4O, which would reduce the overall radiopacity compared to Healos groups incubated overnight (Fig. 4C and 4G).

Tissue engineers should endeavor to form bone with the marrow spaces found anatomically. For instance, the large marrow spaces of the long bone diaphysis would be inappropriate in the calvarium, which contains smaller marrow spaces. Larger marrow spaces were observed between the Healos groups implanted immediately (Fig. 4K and 4O), compared with the Healos groups provided an overnight incubation (Fig. 4C and 4G). The same trend was found when comparing the darkfield area fractions in Fig. 5A. Lower darkfield area fractions corresponded to Healos samples with larger marrow spaces shown in Fig. 4K and 4O, although this effect was not significant here, possibly due to low statistical power (n = 3). Increased remodeling in samples implanted immediately may stem from a greater hematopoietic fraction, the population responsible for producing bone-remodeling osteoclasts. In other words, an overnight incubation could select for a more homogeneous population of mesencyhmal stem cells. Adherence to tissue culture plastic is a well-known selector of mesencyhmal stem cells from the bone marrow, [38], [39] and a second attachment process may purify this population by further removing non-adherent hematopoietic cells. As an indicator of bone resorption, we examined a marker for osteoclast activity using TRAP staining of histological sections from the implants (Fig. S2). We did not find differences in TRAP activity corresponding to delivery with or without an overnight incubation period. However it is possible that differences in osteoclast activity were present at an earlier time point and would not be observed by examining osteoclast activity after such a remodeling event.

An *in vitro* test showed significantly higher loading numbers in Col-HA scaffolds simulating the immediate implantation method compared with overnight incubation (Fig. S3). The discrepancy in cell number between immediate implantation versus overnight incubation samples was due to cell loss to the culture dish when medium was added to the scaffold and when the scaffold was removed from the dish, therefore we expect similar results for Healos scaffolds. It is tempting to speculate that the delivery of a greater number of MSCs in the samples delivered immediately could lead to a more potent hematopoietic niche, resulting in larger marrow spaces in the Healos groups implanted immediately. The evidence supporting this hypothesis stems from the finding that implanted MSCs create a hematopoietic microenvironment that leads to the establishment of marrow cavities. [38] Krebsbach *et al*. noticed that the delivery of higher numbers of MSCs in a thick gelatin vehicle corresponded to the establishment of larger marrow spaces, even though the hematopoietic fraction was very small. [3] Similarly, Liu *et al.*, using cell sorting to generate a homogeneous population of osteoprogenitors, observed larger marrow spaces with a higher cell loading. [40] MSC number positively correlates with bone formation in sites of bone injury. [16] However, MSCs are also a critical component of the native hematopoietic microenvironment and strongly regulate hematopoietic stem cell function through cell-secreted factors (*e.g.* CXCL12) and cell adhesion proteins (*e.g.* Nestin). [41], [42] Further work is needed to examine if the number of seeded MSCs affects the marrow size of a bony implant.

We also investigated if delivering cells to the scaffolds in a complex ECM would enhance *in vivo* bone formation. Several groups have observed beneficial effects of ECM proteins on wound repair and bone formation. [20], [22], [27], [28] ECM proteins can sequester and synergistically amplify signaling factors. [22], [26] Furthermore, a two-component system of collagen-hydroxyapatite is far simpler than the composition of bone. [43] Herein, cells were delivered to scaffolds in a suspension of liquid BME that later gelled upon incubation at 37**°**C. The application of BME gel improved cell penetration (Fig. 6B) in both scaffold types and increased the mean radiopacity of the Col-HA scaffolds (Fig. 3N), only when an overnight incubation period was applied. This could be due to BME gel acting to hold cells in place *in vitro*, since without BME gel, cells can be removed during medium addition after initial seeding and scaffold removal from the culture dish. The evidence to support this hypothesis is that cells depend on proteolytic degradation of the surrounding matrix in order to migrate through BME [44]. This would slow cell migration in BME gel compared with an environment where cells do not require matrix degradation to migrate. Similarly, gels typically have a relatively low permeability, (Collagen gel, k∼10^−13^–10^−12^ m^4^/Ns) [45], which could also reduce cell migration. This suggests that improved cell distribution in scaffolds incubated overnight with BME gel may not be due to enhanced migration, but rather improved cell retention in the scaffolds *in vitro*. The reason that this effect was not observed in the samples implanted immediately could be due to the higher number of delivered cells in groups implanted immediately (Fig. S3). Alternatively, if the effect of BME application on the cell distribution were small for the immediately seeded groups, this would not be noticed here given the small number of animals per group (n = 3).

Some of the Col-HA samples contained modest bone formation (Fig. 4E and 4A) compared with the Healos samples (Fig. 4C and 4G), even when both scaffolds were incubated overnight. The Healos scaffolds had a permeability three orders of magnitude higher than the Col-HA samples used in this study. It has been previously shown by Mitsak *et al*., using polycaprolactone scaffolds and a 3-D printing approach, that increased scaffold permeability led to more bone ingrowth *in vivo*. [46] Permeability is an important mass transport property of a scaffold and therefore has implications for cell survival within a scaffold. Low scaffold permeability and correspondingly limited mass transport may lead to cell death and/or poor migration of invading cells. Limited mass transport may also restrict cell signaling. Therefore scaffold permeability, while not a substitute for cell survival or migration metrics, appears to be a useful physical property of scaffolds that can be measured in advance of *in vivo* or *in vitro* testing and have important implications for *in vivo* bone formation. Future work should evaluate the effect of collagen-HA scaffold permeability on bone formation *in vivo*.

### Conclusions

We examined the effect of an *in vitro* attachment period, the use of BME gel as a cell delivery vehicle, and scaffold type on bone formation *in vivo* with culture-expanded mouse BMSCs. Both scaffold types, with or without an overnight attachment period, were osteogenic *in vivo* following three weeks of implantation. Quantitation of the radiographic images revealed that Col-HA scaffolds implanted immediately had a higher mean radiopacity than Col-HA scaffolds incubated overnight. Similarly, Col-HA scaffolds implanted immediately had higher mean area fractions of donor cells and mineralizing surfaces compared with Col-HA scaffolds incubated overnight. Healos groups implanted immediately had higher area fractions of donor cells compared with Healos scaffolds incubated overnight. The addition of BME gel did not exert a strong effect on the metrics examined here when cells were implanted immediately. However, the use of BME gel as a cell suspension, improved the donor cell distribution in the scaffold when an overnight incubation period was applied. When an *in vitro* test of cell delivery was performed using the Col-HA scaffolds, a higher cell loading was found for the immediate implantation method compared with overnight incubation, with or without BME gel. Due to potential cell loss during overnight incubation, immediate implantation following cell seeding may be a preferable method. These results should be useful when deciding how to deliver cells to a bony defect for optimal cell-based bone tissue engineering.

## Supporting Information

Figure S1 **Radiographic progression of calvarial repairs.** 4 mm critical-size calvarial defects filled with Healos scaffold and neonatal calvarial cells. Radiographs show progression of increase in radiopacity as scaffolds are mineralized over 7, 14, 21, 28, 60, and 100 days after surgery. The rightmost image shows the negative control calvarium, which includes defects filled with the Healos scaffold alone (right hole) and no scaffold or donor cells (left hole).
(TIF)

Figure S2 **TRAP and H&E staining of histological sections.** (Left column) TRAP staining (yellow) superimposed on darkfield images of transverse scaffold sections. (Right column) Hematoxylin and eosin staining of histological sections. Scale bars are 500 µm.
(TIF)

Figure S3 **Comparison of cell number in Col-HA scaffolds.** Cell number was evaluated immediately after loading and following an overnight incubation with and without BME gel. Light red bars indicate 95% confidence intervals and blue bars indicate one standard deviation.
(TIF)
