# Supplementary figures and images for: Effects of Cell-Attachment and Extracellular Matrix on Bone Formation In Vivo in Collagen-Hydroxyapatite Scaffolds
Source: PLoS One. 2014 Oct 16;9(10):e109568. doi: 10.1371/journal.pone.0109568 (PMC4199619; doi:10.1371/journal.pone.0109568)

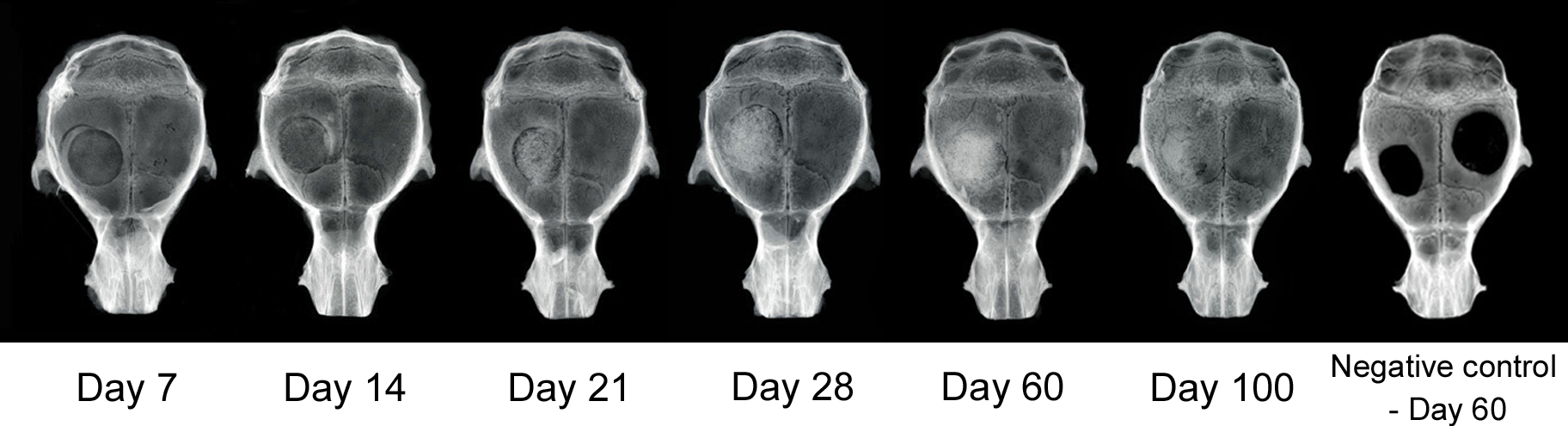

Supplement: Figure S1 — Radiographic progression of calvarial repairs. 4 mm critical-size calvarial defects filled with Healos scaffold and neonatal calvarial cells. Radiographs show progression of increase in radiopacity as scaffolds are mineralized over 7, 14, 21, 28, 60, and 100 days after surgery. The rightmost image shows the negative control calvarium, which includes defects filled with the Healos scaffold alone (right hole) and no scaffold or donor cells (left hole). (TIF) [file pone.0109568.s001.tif]

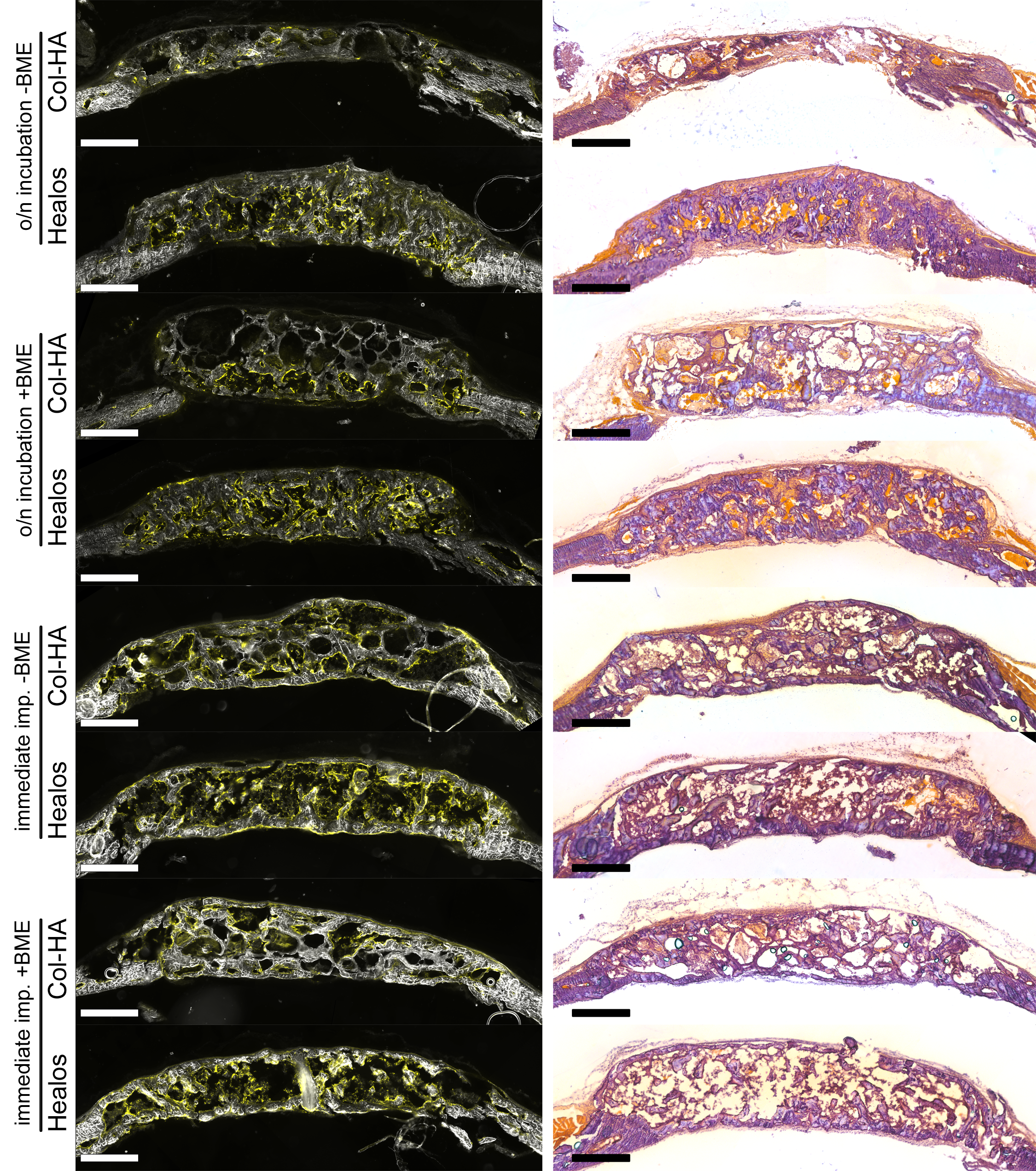

Supplement: Figure S2 — TRAP and H&E staining of histological sections. (Left column) TRAP staining (yellow) superimposed on darkfield images of transverse scaffold sections. (Right column) Hematoxylin and eosin staining of histological sections. Scale bars are 500 µm. (TIF) [file pone.0109568.s002.tif]

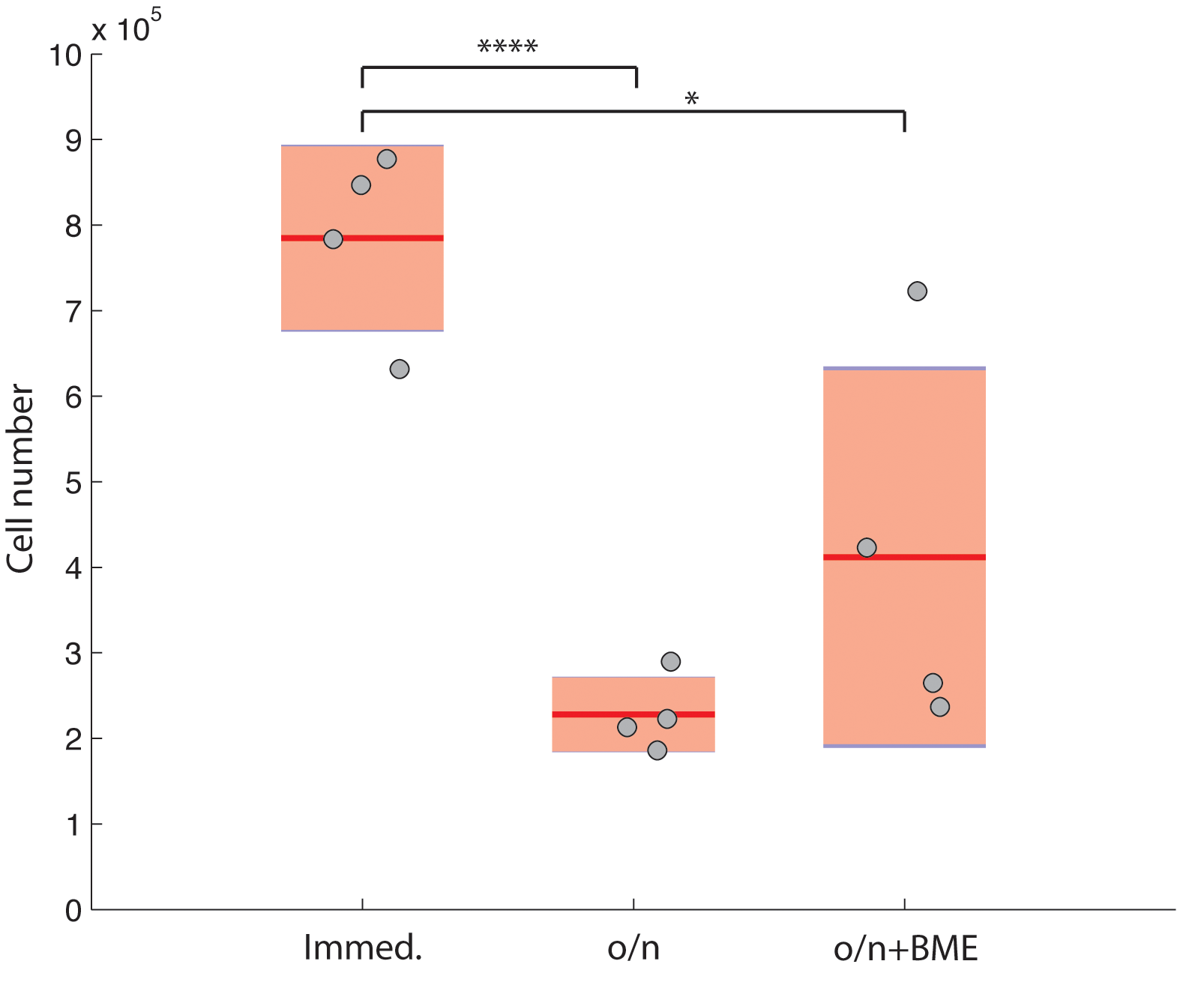

Supplement: Figure S3 — Comparison of cell number in Col-HA scaffolds. Cell number was evaluated immediately after loading and following an overnight incubation with and without BME gel. Light red bars indicate 95% confidence intervals and blue bars indicate one standard deviation. (TIF) [file pone.0109568.s003.tif]
